# Organoids-on-a-chip: microfluidic technology enables culture of organoids with enhanced tissue function and potential for disease modeling

**DOI:** 10.3389/fbioe.2025.1515340

**Published:** 2025-03-11

**Authors:** Lito Papamichail, Lena S. Koch, Devin Veerman, Kerensa Broersen, Andries D. van der Meer

**Affiliations:** ^1^ Department of Surgery, Erasmus MC Transplant Institute, University Medical Center Rotterdam, Rotterdam, Netherlands; ^2^ Department of Internal Medicine, Erasmus MC Transplant Institute, University Medical Center Rotterdam, Rotterdam, Netherlands; ^3^ Applied Stem Cell Technologies, Department of Bioengineering Technologies, University of Twente, Enschede, Netherlands; ^4^ BIOS Lab on a Chip Group, MESA+ Institute for Nanotechnology, University of Twente, Enschede, Netherlands

**Keywords:** organoid, organ-on-a-chip, tissue engineering, biofabrication, drug screening, personalized medicine

## Abstract

Organoids are stem-cell derived tissue structures mimicking specific structural and functional characteristics of human organs. Despite significant advancements in the field over the last decade, challenges like limited long-term functional culture and lack of maturation are hampering the implementation of organoids in biomedical research. Culture of organoids in microfluidic chips is being used to tackle these challenges through dynamic and precise control over the organoid microenvironment. This review highlights the significant breakthroughs that have been made in the innovative field of “organoids-on-chip,” demonstrating how these have contributed to advancing organoid models. We focus on the incorporation of organoids representative for various tissues into chips and discuss the latest findings in multi-organoids-on-chip approaches. Additionally, we examine current limitations and challenges of the field towards the development of reproducible organoids-on-chip systems. Finally, we discuss the potential of organoids-on-chip technology for both *in vitro* and *in vivo* applications.

## 1 Introduction

Organoids are lab-grown, self-organized cellular structures which can be derived from adult, embryonic or induced pluripotent stem cells (hPSCs). They exhibit structural, morphological, and functional characteristics that resemble human organs (Clevers, 2016; Lancaster and Knoblich, 2014b), enabling their use in fundamental and applied research into human physiology and disease (Clevers, 2016; Rossi et al., 2018; Lancaster and Knoblich, 2014b). Until today, several types of organoids have been established, recapitulating the *in vivo* features of various organs, including brain, retina, kidney, intestine, and liver (Qian et al., 2019; Nishinakamura, 2019; Harrison et al., 2021; Almeqdadi et al., 2019).

Despite the advancements that have been made in organoid culture throughout the last years, there are limitations that hamper unlocking the full potential of organoid technology. For example, commonly used organoid culture methods do not permit the accurate recapitulation of the dynamic and complex microenvironment that orchestrates the *in vivo* organ development and maintenance (Sasai, 2013; McCauley and Wells, 2017). The lack of important environmental factors, like biomechanical stimuli and integrated vasculature, inhibits further growth and maturation of organoids into tissues that more closely resemble the native organs (Rossi et al., 2018; Yin et al., 2016). Moreover, common organoid models present high batch-to-batch variability resulting in poor reproducibility that diminishes *in vitro* applications (Hofer and Lutolf, 2021). Researchers developed various approaches to overcome these limitations, including the use of dynamic culture conditions through the application of bioreactors (Qian et al., 2016), vascularization of organoids (Shi et al., 2020), and guided self-organization via several instructive cues (Lancaster et al., 2017). Another promising avenue to address challenges such as reproducibility, environmental cues, maturation and scalability is based on the combination of organoids with microfluidic chip technology.

An organ-on-chip is a three-dimensional (3D) engineered micro- or millisystem used for cell culturing purposes, aiming to recreate the functional units of organs *in vitro* (Esch et al., 2015; Bhatia and Ingber, 2014). The fabricated devices often consist of individually accessible, perfusable chambers of (sub-)millimeter dimensions which enable the incorporation and culture of different cell types and the dynamic control of the culture environment. The complex and dynamic microenvironment involved in *in vivo* organ development, homeostasis and disease can be recapitulated through the incorporation of relevant biochemical and biomechanical cues (Schepers et al., 2016; Huh et al., 2010). By culturing cells in a designed, near-physiological microenvironment, organ-on-chip systems recapitulate functions of both healthy and diseased tissues (Benam et al., 2015).

**FIGURE 1:**
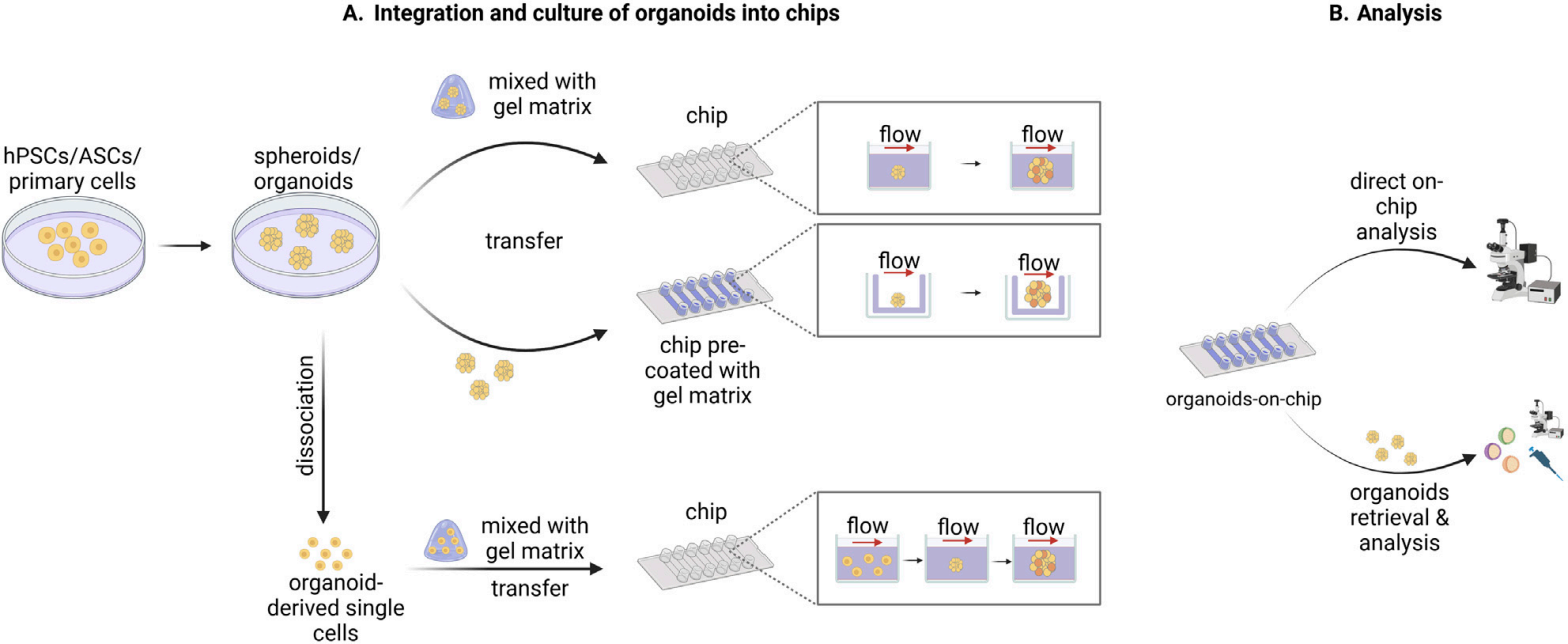
Schematic representation of methods used to integrate, culture, and analyze organoids in chip devices. **(A)** Human pluripotent stem cells (hPSCs), adult stem cells (ASCs), or primary cells are used to form spheroids or organoids according to standard culture protocols. Subsequently, the spheroids/organoids are either mixed with a gel-like matrix and then transferred into the culture chambers of the chip or transferred into a chip pre-coated with a gel-like matrix. Alternatively, the spheroids/organoids are dissociated into single cells which are then mixed with a gel-like matrix and transferred into the culture chambers of the chip. The spheroid/organoid-derived single cells are then organized again into spheroid/organoid structures during the on-chip culture. In all three cases the on-chip spheroid/organoid culture is performed under flow which ensures sufficient medium perfusion. **(B)** The downstream analysis of the organoids cultured into microfluidic chips can be conducted either directly on chip or after the retrieval of organoids from the chip depending on the design of the system used and the nature of the subsequent analysis. Created in BioRender. Papamichail, L. (2025) https://BioRender.com/a07j366.

In contrast to the typical on-chip cultures based on two-dimensional (2D) monolayers, organ-on-chip platforms have been used for culturing 3D tissue constructs, like cell aggregates, spheroids and organoids. These 3D tissues can be integrated into microfluidic chips in multiple ways (Figure 1). In one approach, cell aggregates or organoids are first formed according to standard culture protocols, after which they are mixed with a gel-based matrix and transferred into the culture chambers of the chip. The gelation of the matrix leads to the immobilization of the tissue in the chamber (Wang et al., 2018a; Karzbrun et al., 2018; Berger et al., 2018). In other strategies, the pre-formed organoids are directly seeded in a platform that has been previously coated with gel-like matrix permitting their adhesion on top of the gel surface (Homan et al., 2019; Lee et al., 2021). Another approach is based on the seeding of organoid-derived single cells in the platform and their subsequent on-chip assembly into organoids (Wang et al., 2020). In all cases, during on-chip culture, medium perfusion is ensured through the application of defined flow patterns generated by pump systems. Analysis of organoids cultured in chip platforms can be performed either directly on the microfluidic device or after retrieving the organoids from the chip through mechanical dissociation (Figure 1; Park et al., 2019).

**FIGURE 2:**
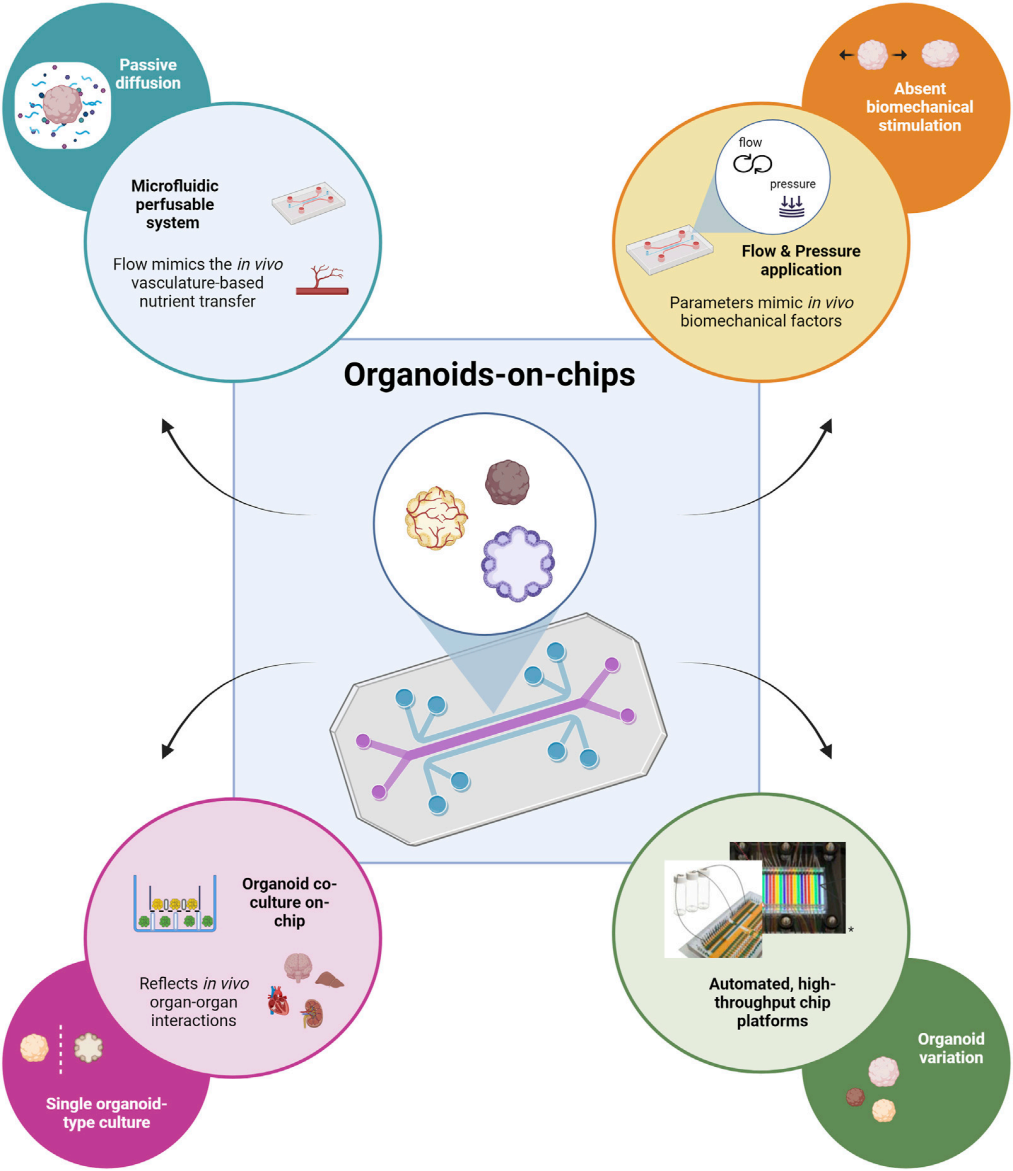
Schematic representation depicting how organoid-on-chip technology can contribute to addressing the major limitations of typical organoid culture. The flow of culture medium through the microfluidic perfusable network in the chip mimics *in vivo* vasculature-based nutrient transfer; addresses the limitation of passive nutrient diffusion on the extended culture and growth of organoids (top left). The application of flow and pressure through the chip device recapitulates *in vivo* biomechanical factors; addresses the lack of biomechanical stimulation important for the further organoid maturation (top right). The possibility of co-culturing different types of organoids provides the opportunity to capture *in vivo* organ-organ interactions; it overcomes the lack of organ-level communication during the standard single-type organoid culture (bottom left). Platforms that permit automated, high-throughput on-chip culture can be used to reduce organoid variability observed for standard organoid culture methods (adapted from Schuster et al., Nat Commun, 2020, CC BY 4.0 (Schuster et al., 2020)) (bottom right). Created in BioRender. Papamichail, L. (2025) https://BioRender.com/o40q718.

Organ-on-a-chip technology can be used to overcome some of the major limitations of conventional organoid culture as reviewed by Park et al. (2019), and more recently by Zhao et al. (2024). First, chip devices offer the possibility to control the culture microenvironment. Standard organoid culture methods depend on passive diffusion for the exchange of oxygen, nutrients and waste products which does not permit extended organoid growth as it leads to the development of hypoxic cores or cell death. *In vivo*, vascular formation supports organ development mediating the nutrient and waste exchanging processes (Weinstein, 1999). The integration of a perfusable microfluidic system to mimic vasculature function can overcome the diffusion limitations (Figure 2, top left; Kim et al., 2013; Kolesky et al., 2016). Another important environmental factor missing in conventional organoid culture is biomechanical stimulation. Mechanical forces hold a crucial role in developmental and physiological processes and can be recapitulated by chip platforms through application of flow and pressure (Figure 2, top right; Vining and Mooney, 2017). Micro- and milli-engineered fluidic devices, manipulating fluidic flows from micrometers to millimeters respectively (Chen et al., 2021), also offer the possibility to increase the number of organoids that can be cultured and analyzed simultaneously, thus enabling organoid screening, selection, and manipulation at a high-throughput level. Finally, chip devices permit the integration of tissue-tissue interactions which play a central role during *in vivo* organ development. The co-culture of different organoid types in a microfluidic platform can capture important aspects of the complex *in vivo* organ-organ communication (Figure 2, bottom left; Drost and Clevers, 2017). Organ-on-chip technology can provide automated and high-throughput culture setups which aid in targeting the significant variability observed in conventional organoid generation (Drost and Clevers, 2017; Yin et al., 2016). Advanced automated platforms that allow precise control of microgeometries and medium refreshment can be used to avoid inconsistencies between experimental parameters imparted by manual manipulation (Figure 2, bottom right; Drost and Clevers, 2017).

Here, we review the latest breakthroughs in the incorporation of stem cell-derived organoids into organ-on-chip platforms to enhance functionality and disease modeling. We compare advancements achieved with organoid-on-chip cultures to their traditionally cultured counterparts to assess how microfluidic integration addresses key limitations of conventional cultures such as improving organoid maturity and reproducibility and creating more physiologically relevant models. Furthermore, with the recognition that organ-organ interaction critically regulates organ functionality, we discuss reported multi-organoid-on-chip strategies. We will discuss current biological challenges, such as inherent organoid variability, vascularization and environmental cues, and technical challenges, such as reproducibility and scalability. Finally, we will outline future directions of the field of organoids-on-chip.

## 2 Organoids-on-chip

### 2.1 Brain organoids

Brain organoids generated from pluripotent stem cells recapitulate architectural and functional characteristics of the human brain. They exhibit features that mimic brain development with the formation of complex, organized and regionalized structures. These organoids have provided significant insights into human brain development and neurological disorders (Kadoshima et al., 2013; Lancaster et al., 2013). However, the extended cultivation and maturation of cerebral organoids is hindered due to insufficient nutrient and oxygen supply often resulting in necrotic core formation (Qian et al., 2016; Lancaster et al., 2013; Lancaster and Knoblich, 2014a). In addition, conventional culture methods lack essential environmental cues, such as biomechanical stimulation, and matrix-cell and cell-cell interactions, only permitting the formation of organoids representing early brain developmental stages (Camp et al., 2015). To model later developmental stages, organoids require an extended cultivation period of six to 9 months (Quadrato et al., 2017). Thus, there is an urgent need for advancements in generating and culturing more functionally and structurally mature brain organoids to address these limitations.

#### 2.1.1 Proof-of-concept brain-organoid-on-chip cultures reveal enhanced neural development

Lancaster et al. were the first to incorporate brain organoids into microfluidic chips and examined the feasibility of this approach. In the following examples, the methodology for incorporating brain organoids into the chip system was consistently guided by a specific approach. Adapting the Lancaster protocol for unguided cerebral differentiation (Lancaster et al., 2013), embryoid bodies (EBs) were initially generated. Once successful neuroectoderm induction was achieved by culture day 11, EBs were transferred onto the chip, initiating neural differentiation. The on-chip culture and differentiation continued until approximately day 30. Subsequently, the efficacy of the incorporation was assessed by evaluating the extent of neural differentiation of EBs into cerebral organoids. This assessment involved examining the expression of key neural markers and the structure and spatial organization of the developed organoids.

Wang et al. presented one of the pioneering brain organoids-on-chip systems (Wang et al., 2018a). The authors reported critical aspects of successful neural differentiation within the organoids evidenced by the expression of relevant markers. Specifically, results showcased an increasing expression of neural progenitor markers Nestin and SOX2 over the culture period combined with a concurrent decrease of pluripotency marker OCT4. Additionally, organoids also expressed markers of early (TUJ1), preplate (TBR1) and deep-layer (CTIP2) neurons. Comparatively, organoids cultivated on-chip showed higher expression levels and more defined structural organization of TUJ1 and SOX2 in contrast to those grown in conventional well plates. Apical surface marker CD133 expression patterns suggested organoids forming brain ventricle-like fluid-filled structures, with on-chip organoids exhibiting larger cavities than well-plate counterparts. Regionalization was also observed through the expression of genes associated with specific brain regions: PAX6 and FOXG1 indicating forebrain development, and PAX2 and ISL1 signifying hindbrain patterning. This regionalization became more distinct as the organoids matured (Wang et al., 2018a). The above findings are consistent with other reports generally focusing on the same set of markers and spatial organization specifying neural differentiation on chip (Karzbrun et al., 2018; Wang et al., 2018b). Notably, during the initial weeks of on-chip culture, rosette-like structures were identified, subsequently evolving into larger and more complex organoids at later stages (Wang et al., 2018a; Berger et al., 2018). Cortical formation and organization were indicated through the development of an initial cortex structure consisting of both basal and deep neural layers. Collectively, the aforementioned studies affirm the successful integration of brain organoids into microfluidic chips, resulting in a system that enables more efficient neural and early cortex development compared to conventional well plate-based culture methods.

#### 2.1.2 Fluid flow control enhances brain organoid differentiation and functionality

**FIGURE 3:**
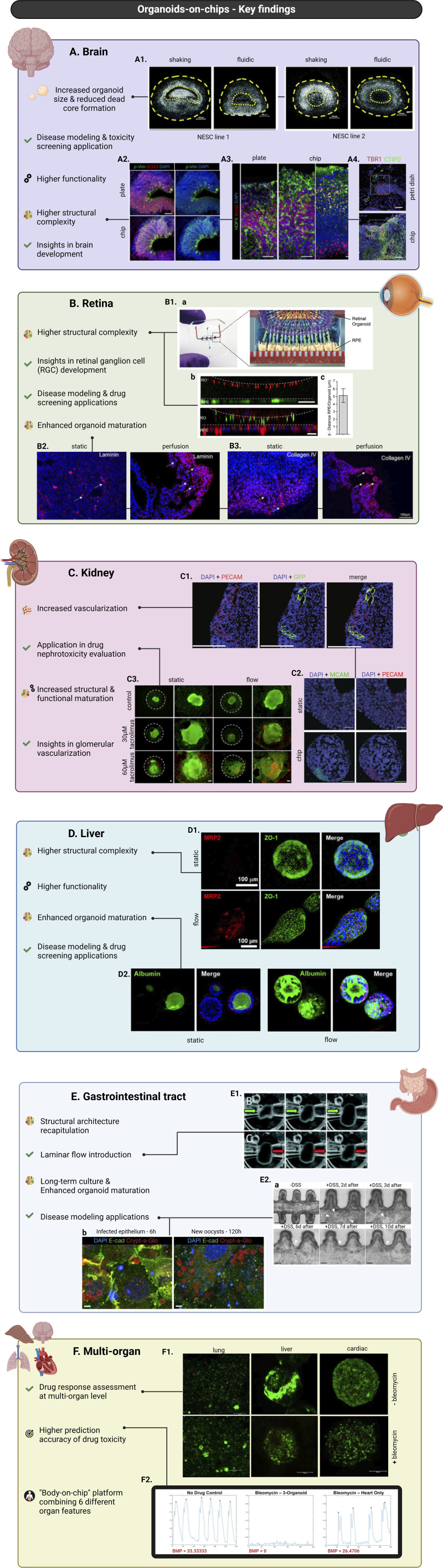
Major advances in organoids-on-chip technology, focusing on the incorporation of brain **(A)**, retinal **(B)**, kidney **(C)**, liver **(D)**, gastrointestinal tract **(E)**, and multi-organoids **(F)** into chips. Brain organoids cultured into chips overall present increased diameter size and higher structural complexity and functionality. Applications of brain organoids-on-chip systems led to insights into mechanisms underlying brain development and enabled modelling of brain developmental disorders and toxicity screening studies **(A)**. Nuclei staining of hMOs derived from two different neuroepithelial stem cell lines (NESC) showing reduced necrotic core formation under controlled fluidic conditions compared to common shaking culture (scale bars = 200 μm, adapted from Berger et al., Lab on a Chip, 2018, CC BY 3.0 (Berger et al., 2018)) **(A1)**. Immunostaining of neural progenitor marker SOX2 and radial glia marker phosphorylated vimentin (p-Vim) show increased glia generation in chip-culture of brain organoids compared to plate-culture, indicating increased organoid maturation (scale bar = 50 μm, adapted from Cho et al., Nat Commun, 2021, CC BY 4.0 (Cho et al., 2021)) **(A2)**. Immunostaining of basal layer marker SOX2 showing increased cortical organization and higher structural complexity of brain organoids cultured into chip versus on well-plate (scale bars = 50 μm, adapted from Cho et al., Nat Commun, 2021, CC BY 4.0 (Cho et al., 2021)) **(A3)**. Immunostaining for early-born neuron marker CTIP2 and preplate marker TBR1 show enhanced spatial cortical neuronal organization of organoids cultured in chips versus Petri dishes (scale bars = 50 μm, adapted from Wang et al., RSC Advances, 2018, CC BY NC 3.0 DEED (Wang et al., 2018a)) **(A4)**. Retinal organoids cultured into chips demonstrate higher structural complexity and enhanced maturation. Applications of retinal organoids-on-chip systems led to insights into mechanisms underlying retinal ganglion cell (RGC) development and enabled disease modelling of retinal diseases and drug screening studies **(B)**. Schematic representation of organ-on-chip system used for the co-culture of retinal organoids (ROs) with RPE cells **(B1a)**. Live-cell imaging shows the interaction between pJG-IRBP-GFP viral vector-labelled RPE cells and PNA lectin-Alexa Fluor 568-labelled ROs (scale bar = 40 μm) (**B1b**, top). Immunostaining of rhodopsin (green) and phalloidin (red) reveals a defined interaction of ROs and RPE cells (scale bar = 40 μm) (**B1b**, bottom). Graph depicting distance between GFP-labelled RPE cells and lectin-labelled segment tips (figures **B1a–c** adapted from Achberger et al., eLife, 2019, CC BY 4.0 (Achberger et al., 2019)) **(B1c)**. Immunostaining of laminin (red) showing increased expression in ROs cultured under perfusion compared to static conditions (scale bars = 100 μm, adapted from Su et al., Front. Bioeng. Biotechnol., 2022, CC BY 4.0 (Su et al., 2022)) **(B2)**. Immunostaining of collagen IV (red) showing increased expression in ROs cultured under perfusion compared to static conditions (scale bars = 100 μm, adapted from Su et al., Front. Bioeng. Biotechnol., 2022, CC BY 4.0 (Su et al., 2022)) **(B3)**. Kidney organoids cultured into chips demonstrate increased structural and functional maturation and higher degree of vascularization. Applications of kidney organoids-on-chips resulted in insights into glomerular vascularization and facilitated drug nephrotoxicity evaluation studies **(C)**. Immunostaining for nuclei (DAPI/blue), HUVECs (PECAM/red^+^, GFP^+^), and native ECs (PECAM/red^+^, GFP^−^) showing vascular structures combinatorically formed by both HUVECs and native ECs (scale bars = 200 μm, adapted from Bas-Cristóbal Menéndez, A., Du, Z., van den Bosch, T.P.P. et al., Sci Rep, 2022, CC BY 4.0 (Bas-Cristóbal Menéndez et al., 2022)) **(C1)**. Immunostaining of nuclei (DAPI/blue), MCAM (green), and PECAM (red) revealing increased MCAM+ and PECAM + areas during on-chip conditions compared to static (scale bars = 200 μm, adapted from Bas-Cristóbal Menéndez, A., Du, Z., van den Bosch, T.P.P. et al., Sci Rep, 2022, CC BY 4.0 (Bas-Cristóbal Menéndez et al., 2022)) **(C2)**. Live (green)/dead (red) staining of untreated (control) kidney organoids and kidney organoids treated with tacrolimus shows increased tacrolimus-mediated cell death of kidney organoids cultured under flow versus static conditions (scale bars = 100 μm, adapted from Lee. H. N. et al., Nano Convergence, 2021, CC BY 4.0 (Lee et al., 2021)) **(C3)**. Liver organoids cultured into chips demonstrate higher structural complexity and enhanced maturation and functionality. Applications of liver organoids-on-chip systems include disease modelling and drug screening studies **(D)**. Immunofluorescent staining for MRP2 (red) and ZO-1 (green) showing more prominent and structured expression in culture under flow compared to static (scale bars = 100 μm, adapted from Byeon et al., Front. Cell Dev. Biol., 2024, CC BY 4.0 (Byeon et al., 2024)) **(D1)**. Immunofluorescent staining for albumin (green) revealing higher expression in culture under flow compared to static (scale bars = 100 μm, adapted from Byeon et al., Front. Cell Dev. Biol., 2024, CC BY 4.0 (Byeon et al., 2024)) **(D2)**. Gastrointestinal organoids cultured into chips show better recapitulation of the *in vivo* structural architecture, prolonged culture possibilities, enhanced organoid maturation and permit introduction of laminar flow. Applications of gastrointestinal organoids-on-chip systems include modelling of biology and disease with a focus on host-microorganism interactions and regeneration processes **(E)**. Demonstration of laminar flow introduction in intestinal organoids showing expansion and contraction of the organoids in accordance with flow application and/or direction conditions (adapted from Sidar et al., Lab-on-a-Chip, 2019, CC BY-NC 3.0 (Sidar et al., 2019) **(E1)**. Time-course images of DSS-injured epithelium showing epithelial regeneration 10 days post-injury (Reprinted by permission from Nikolaev, M., Mitrofanova, O., Broguiere, N. et al., Copyright 2020, Springer Nature (Nikolaev et al., 2020)) **(E2a)**. Immunostaining of epithelial intestinal cells (nuclei/DAPI-blue, actin filaments/E-cadherin-green) and parasite *C. parvum* (oocyst outer walls/Crypt-a-Glo-red) demonstrating the infection progression of the intestinal epithelium (Reprinted by permission from Nikolaev, M., Mitrofanova, O., Broguiere, N. et al., Copyright 2020, Springer Nature (Nikolaev et al., 2020)) **(E2b)**. Key findings in multi-organoids-on-chip systems enabled the assessment of drug response at a multi-organ level, the development of a single chip system combining features of six different organs and the design of platforms with higher prediction accuracy of drug toxicity **(F)**. Live/dead (green/red) staining of co-cultured lung, liver, and cardiac constructs with and without bleomycin treatment demonstrating the application of multi-organoid-on-chip platforms on drug response assessment at a multi-organ level (adapted from Skardal et al., Scientific Reports, 2017, CC BY 4.0 (Skardal et al., 2017)) **(F1)**. Plots depicting beating of cardiac organoids in different experimental conditions revealing a substantially more prominent effect of bleomycin in on-chip co-culture conditions (three-organoids) compared to mono-culture ones (heart only) (adapted from Skardal et al., Scientific Reports, 2017, CC BY 4.0 (Skardal et al., 2017)) **(F2)**. Created in BioRender. Papamichail, L. (2025) https://BioRender.com/d31m438 **(A)**, https://BioRender.com/d31m438 **(B)**, https://BioRender.com/d31m438 **(C)**.

**FIGURE 4:**
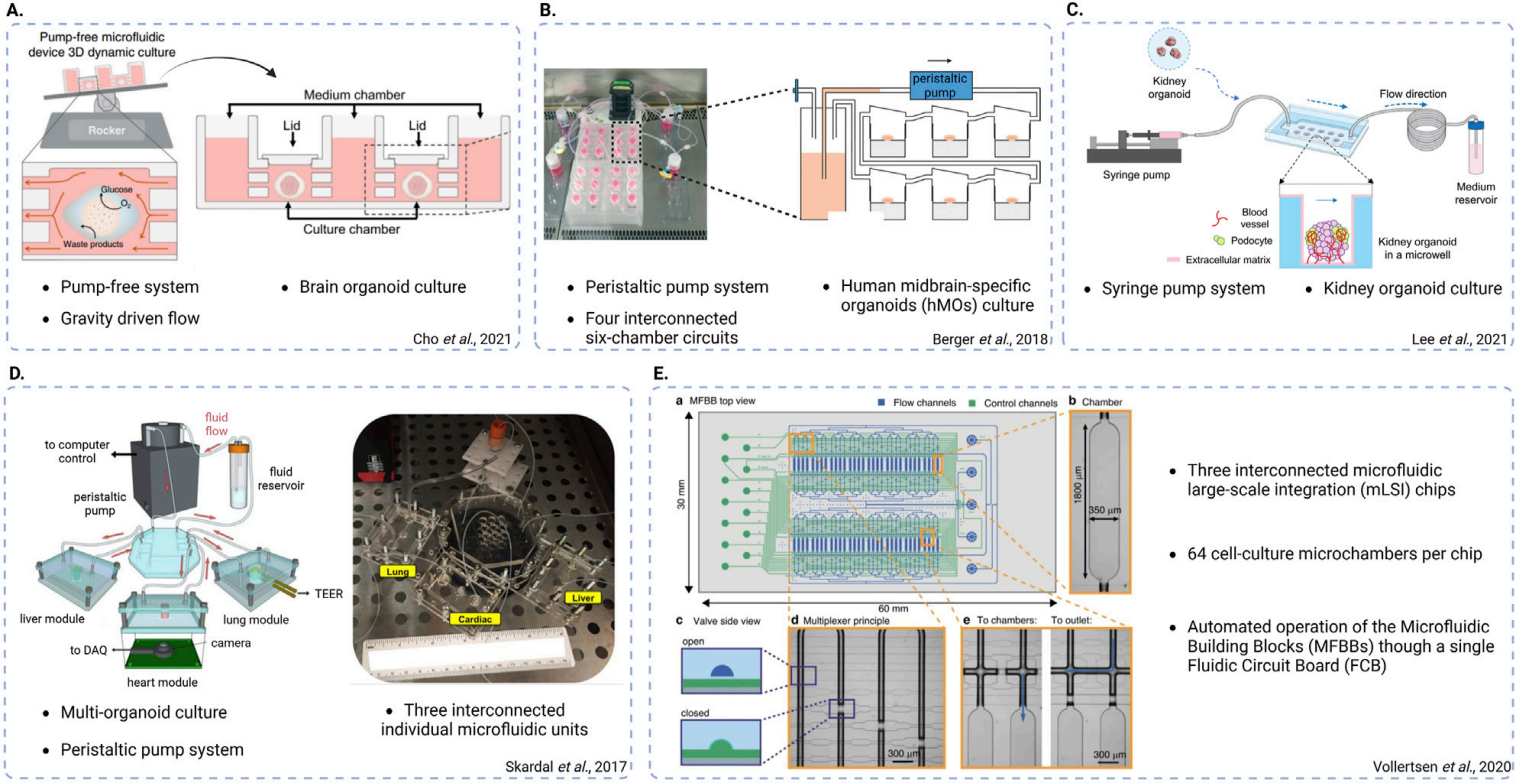
Schematic representations of different chip system designs. **(A)** Pump-free, gravity-driven fluidic system used to culture brain organoids and mimic bi-directional cerebrospinal flow *in vitro* (adapted from Cho et al., Nat Commun, 2021, CC BY 4.0 (Cho et al., 2021). **(B)** Peristaltic pump-based system used to culture human midbrain-specific organoids (hMOs), featuring four interconnected six-chamber circuits (adapted from Berger et al., Lab on a Chip, 2018, CC BY 3.0 (Berger et al., 2018)). **(C)** Syringe pump-based system used to culture kidney organoids and promote vascularization (adapted from Lee. H. N. et al., Nano Convergence, 2021, CC BY 4.0 (Lee et al., 2021)). **(D)** Peristaltic pump-based, multi-organoid system featuring three interconnected individual fluidic units each one used for the integration of a different organoid type (adapted from Skardal et al., Scientific Reports, 2017, CC BY 4.0 (Skardal et al., 2017)). **(E)** System featuring three interconnected microfluidic large-scale integration (mLSI) chips, each one consisting of 64 cell culture microchambers. The design enables automatic operation of the microfluidic building blocks (MFBBs) through a single fluidic circuit board (FCB) (adapted from Vollertsen et al., Microsystems and Nanoengineering, 2020 CC BY 4.0 (Vollertsen et al., 2020)). Created in BioRender. Papamichail, L. (2025) https://BioRender.com/l34j257.

Following these “proof-of-concept” studies, more recent research has shifted its focus towards comparing brain organoids-on-chip systems with conventional brain organoid culture methods (Cho et al., 2021; Berger et al., 2018). For instance, Cho et al. utilized a microfluidic device that allowed precise control of fluid flow mimicking bi-directional cerebrospinal flow *in vivo*. After neuroectoderm induction, the organoids were embedded into hydrogels based on brain extracellular matrix (BEM) and subsequently transferred to the microfluidic device for culturing. When compared to organoids cultured in static BEM hydrogels, the BEM-chip organoids exhibited notable advantages. The microfluidic setup facilitated an increased glucose diffusion rate throughout the organoids and higher and more homogeneous intra-organoid oxygen levels. Consequently, chip organoids exhibited enhanced proliferation, reduced heterogeneity and apoptosis as well as larger volume size and extended culture lifespan (up to 120 days). Chip organoids also presented greater structural complexity and promotion of corticogenesis indicated by various characteristics (Figures 3A2–A4). Notably, organoids developed under fluidic conditions showed thicker zones of deep and upper-layer neurons and an enrichment in cell types involved in cortex formation. Transcriptomic analysis revealed increased expression of genes involved in neuronal differentiation and migration. The overall electrophysiological functionality of chip-cultured organoids was significantly improved compared to those cultured in traditional plates. Most interestingly, chip organoids exclusively exhibited postsynaptic currents, implying the formation of a postsynaptic compartment (Cho et al., 2021) (Figure 4A). Another study by Berger et al. incorporated human midbrain-specific organoids (hMOs) into a millifluidic device and compared them with hMOs cultured in shaking well plates. Here, the millifluidic system QuasiVivo900^®^ (Kirkstall, UK) was used in conjunction with hMOs that were placed in each of the chambers (Figure 4B). The millifluidic chip provided better controlled fluid flow compared to common shaking conditions leading to a reduced necrotic core size within the organoids (Figure 3A1). Through computational analysis, it was determined that the fluidic system increased the oxygen concentration in the organoid, resulting in a reduced cell death rate. In addition, midbrain dopaminergic (mDA) neuronal differentiation was more pronounced in the fluidic setup as evidenced (Berger et al., 2018).

Ao et al. addressed the challenge of enhancing organoid perfusion by developing a 3D-printed hollow mesh scaffold that supported the growth of cerebral forebrain organoids in a tubular shape (Ao et al., 2021). The device was compatible with multi-well formats, including 24-, 96-, and even 384-well plates, enabling higher-throughput organoid culture. Combined with a rocking platform, this design allowed constant medium and oxygen perfusion through the organoid, effectively preventing hypoxic core formation during a culture period of approximately 40 days. This was substantiated by the absence of hypoxia dye signal and significantly lower expression of hypoxia marker HIF1a compared to conventional growth conditions. The tubular culture system furthermore facilitated the development of a more evenly distributed and size-consistent ventricular/subventricular zone (VZ/SVZ) while promoting neural progenitor proliferation, indicated by significantly higher percentage of EdU + cells. Additionally, it supported greater neural fate commitment and differentiation when directly compared to conventionally grown forebrain organoids. As a proof-of-concept, the authors applied the tubular organoid system to model microglia-mediated neuroinflammation and possible treatment strategies. Notably, the activated microglia integrated in the tubular organoid system exhibited stronger cytokine responses compared to conventional 2D microglia cultures. This showed first steps towards establishing immune-competent cerebral organoids on chip (Ao et al., 2021).

#### 2.1.3 Higher-throughput and automated platforms for cerebral organoid culture

In the studies discussed above, EB generation and neuroectoderm induction were performed in commercial well plates, requiring manual transfer of EBs to individual microfluidic devices for further differentiation, maturation and application. Zhu et al. streamlined this process by designing a microfluidic platform featuring micropillar arrays that facilitated EB formation as well as their subsequent differentiation, and maturation (Zhu et al., 2017). The platform enabled higher-throughput organoid generation of up to 160 organoids simultaneously and improved organoid homogeneity by optimizing the micropillar spacing. Cerebral organoids produced on this platform in the “proof-of-concept”-study expressed neural differentiation and brain regionalization markers, and by day 40, exhibited a defined cortical layer architecture and ventricular formation.

The platform was subsequently utilized for a study series on the effects of risk substances and maternal illnesses on fetal brain development. Yin et al., applied the microarray platform to model the effects of prenatal cadmium exposure, demonstrating that cadmium lead to impaired neurogenesis in the developing brain through increased apoptosis, disrupted neural differentiation and abnormal brain regionalization (Yin et al., 2018). Similarly, Cui et al. investigated prenatal exposure to valproic acid (VPA), a drug frequently used in the treatment of epilepsy, using cortical organoids. VPA exposure induced neurodevelopmental abnormalities such as disrupted neural progenitor formation and neuronal differentiation, altered gene expression and impaired brain regionalization. These findings highlighted autism-related risks associated with VPA exposure due to the resemblance to expression patterns and developmental abnormalities of autism-derived organoids and patient samples (Cui et al., 2020). In the most recent application, the microarray chip was used to assess the impact of maternal breast cancer on fetal neurodevelopment (Cui et al., 2022). Exposure to breast cancer-derived exosomes impaired neurogenesis marked by upregulated stemness markers and enrichment of carcinogenesis-related pathways, emphasizing the detrimental impact of maternal breast cancer on the neurodevelopment of the fetus (Cui et al., 2022). In this study, healthy organoids were cultured up to 70 days showing the efficient neural induction, forebrain regionalization and cortical structure formation in the native organoids. Yet, issues like uncontrolled fluid flow, oxygen diffusion limitations, and necrotic core formation remain challenges in longer-term cultures.

Ao et al. addressed oxygen diffusion limitations by introducing a liquid-air interface in their microfluidic device to enhance oxygenation of the medium, reducing necrotic core formation in cerebral organoids (Ao et al., 2020). The microfluidic chip featured individual compartments where up to 169 organoids rested on metal mesh above a medium supply channel. The generated cerebral organoids, viable for up to 90 days, expressed relevant neurodevelopmental markers, brain regionalization, and electrophysiological functionality. Hypoxic core formation was reduced compared to standard culture methods and reproducibility in homogenous organoid size was improved, as organoids were confined to 2 mm by the compartment architecture. Using this platform, Ao et al. investigated tetrahydrocannabinol (THC) neurotoxic effect on the prenatal brain development. Cannabis use during pregnancy has been linked to increased risk for psychiatric disorders in offspring. Adverse effects of THC in the developing brain had previously been shown in rodent models but not in human studies. Ao et al. showed that THC exposure impaired neural maturation reduced neurite outgrowth and disturbed the spontaneous firing rate in human brain organoids (Ao et al., 2020).

Another venture to optimize cerebral organoid formation in a multiplex setting was proposed by Seiler et al. (Seiler et al., 2022). The authors presented an automated microfluidic system to minimize manual handling steps in organoid culture. The PDMS chip, with a 24-well plate footprint, featured individual inlets and outlets for medium changes at tightly controlled rates. The optically clear device supported live imaging during incubation enabling continuous monitoring over time. EBs were transferred to the device on day 12 and further differentiated and grown. The cerebral organoids were comparable to conventionally grown organoids in terms of growth, size and relevant marker expression but showed a significant downregulation of ER stress markers that were upregulated during conventional organoid differentiation, potentially leading to an aberrant cerebral subtype specification.

Recently, Zhu et al. utilized microfluidic technology to create human brain assembloids, aiming to achieve not only higher throughput production but also greater complexity and representativeness of multiple regions of the brain (Zhu et al., 2023). They first encapsulated PCSs via microfluidic electrospray in hydrogel microcapsules to form EB, which were then further differentiated towards cortical, hippocampal and thalamic organoids. Subsequently, a multilayer microfluidic chip with complementary micropillar-microhole array system was used to organize the organoids into defined patterns for assembly. After removing the microcapsule shell, the organoids fused to assembloids with functional connectivity characterized by synapse formation, and active neural migration and interaction (Zhu et al., 2023).

#### 2.1.4 Vascularization of brain organoids suggests accelerated maturation

In a study by Salmon et al., on-chip culture was used to induce vascularization of brain organoids (Salmon et al., 2022). The authors presented a preliminary effort to mimic the *in vivo* spatial orientation and temporal synchronization orchestrating the early vasculature-tissue co-development. For this purpose, cerebral organoids were either mono-cultured in the chip or co-cultured with hPSC-derived pericytes and endothelial cells. Upon co-culture, pericytes and endothelial cells exhibited sprouting towards the cerebral organoid, thereby establishing physical interactions and creating a neurovascular-like unit. Interestingly, a comparison of mature and immature neuron markers expression patterns between mono- and co-culture conditions suggested accelerated cerebral organoid maturation facilitated by the vascularization.

#### 2.1.5 From fundamental insights to disease modeling applications

Some of the studies discussed above have also provided preliminary findings highlighting the potential applications of the reported brain organoids-on-chip systems. Karzbrun et al. illustrated the utility of their brain organoid-on-chip system for elucidating the underlying mechanisms steering gyrification during brain development. This demonstrated the relevance of the chip systems for studying biological and physical processes involved in human brain development (Karzbrun et al., 2018). Another promising application of organoid-on-chip technology was presented by Wang et al. who applied the platform for disease modeling and toxicity screening, specifically for modeling prenatal nicotine exposure (Wang et al., 2018b). The potential for disease modeling was also suggested in the work of Berger et al. which demonstrated the use of hMOs-on-chip for modeling Parkinson’s disease (Berger et al., 2018).

### 2.2 Retinal organoids

The study of hPSC-derived retinal organoids (ROs) increased in popularity over the last decade. ROs recapitulate the complex architecture of the tissue found in the human retina (Li et al., 2021). Recently, the focus of RO research is towards developing ROs that contain genetic defects to recapitulate certain inherent retinal diseases, such as retinitis pigmentosa (Su et al., 2022).

However, as with all organoids, ROs come with certain drawbacks such as the lack of vascularization, formation of a hypoxic core and limited maturation (Andrews and Kriegstein, 2022). A specific drawback of the currently described ROs is the absence of a monolayer of retinal pigment epithelium (RPE) which is in direct contact with the photoreceptors *in vivo* and plays a key role in the visual cycle (Achberger et al., 2019).

Achberger et al. were the first to successfully incorporate ROs and RPE cells on chip (Achberger et al., 2019). The open-top microfluidic chip consisted of a channel for medium perfusion which was separated from the RPEs using a semi-permeable membrane (Figure 3B1a). The ROs were placed on top of the RPE cells which were cultured on top of the membrane. In this way, both cell types remained in close contact with each other (Figure 3B1b–c). This enhanced the development and maturation of the ROs, as shown by the invasion of rhodopsin in the hydrogel between both structures and the increased formation of photoreceptor inner and outer segment-like structures. Functionality of the RPE cells was demonstrated by phagocytosis of photoreceptor outer segments (Achberger et al., 2019). Another study demonstrating the feasibility of culturing ROs on-chip was reported by Xue et al. (Xue et al., 2021). In this study, a micro-millifluidic bioreactor was generated and optimized for mass transfer efficiency and media concentration uniformity among the chambers in the chip. ROs at various differentiation stages were manually added to and trapped in the chambers of the 30-chambered chip and cultured for more than 1 month. Although there was no significant improvement in the differentiation and maturation of the ROs as compared to static culture, Xue and coworkers did show that the chip produced ROs of comparable quality while reducing the labour intensity (Xue et al., 2021).

#### 2.2.1 Fluid flow control enhances retinal ganglion cell differentiation

More recently, Gong et al. showed enhanced retinal ganglion cell (RGC) development in ROs using a microfluidic chip with controllable perfusion (Gong et al., 2023). They produced a polydimethylsiloxane (PDMS)-based microfluidic chip which consisted of two layers in which a polyethylene membrane was sandwiched, thereby reducing fluid shear stress exerted on the ROs. The bottom layers contained microwells in which early-stage hPSC-derived ROs were placed and could be subjected to various levels of fluidic shear stress. The authors evaluated the growth of ROs on the chip and compared that to standard dish culture. It was shown that the retinal progenitor cells within the ROs were expanded using low levels of shear stress. Besides, it appeared that the chip promoted RGC development and that there were more RGC subtypes found in the chip as compared to dish culture. Furthermore, axonal outgrowth was more pronounced and RNA sequencing assays showed that RGC development was mediated by activating voltage-gated ion channels and enhanced expression of extracellular matrix proteins.

Overall, this study demonstrated the power of using perfusion microfluidic chips in the development and maturation of ROs. Yet, it is still unclear how the ROs will develop and mature beyond 39 days when maintained in such microfluidic systems (Gong et al., 2023).

Moreover, recent efforts by Drabbe et al. demonstrated the ability to create and maintain a physiological oxygen gradient in a high-throughput Retina-on-Chip device (Drabbe et al., 2025). The PDMS-free microfluidic device contained 55 wells, in which retinal organoids up to 1000 µm were trapped, connected to a fluidic medium channel, On the bottom a thin chamber, filled with oxygen scavenger solution, was separated from the organoids by a perfluoroalkoxy alkane membrane. All materials were chosen to limit the oxygen permeability to allow for optimal control of oxygen levels within the chip. After validation of the device using COMSOL Multiphysics^®^ and oxygen probe measurements, six-week-old ROs were cultured in the chip with and without oxygen gradient which were then compared to ROs in a dish. Interestingly, the expression of RBPMS, a RGC specific marker, was significantly increased in the chip with the oxygen gradient as compared to the other conditions although there were great differences between individual data points. Although Drabbe and colleagues were the first to incorporate fluidic oxygen control in a novel retina-on-chip system, more and different readouts, such as gene expression analysis, would improve their message. Besides, the effect of the oxygen gradient on the whole differentiation process of retinal organoids would be interesting to study as well (Drabbe et al., 2025).

#### 2.2.2 Applications of retinal organoids-on-chip models in drug testing and gene therapy

Some possible applications of models with on-chip culture of ROs in drug testing and disease modelling have been reported as well. For example, to demonstrate the potential of the model for drug testing purposes, Achberger and colleagues investigated the toxic effects on the retina when administering chloroquine and gentamicin, an anti-malaria drug and an antibiotic, respectively. According to the authors, the experiments demonstrated successful recapitulation of the toxic effects of both drugs and the protective function of the RPE barrier (Achberger et al., 2019). More recently, Achberger and colleagues took the previously reported retina-on-chip to the next level by demonstrating gene therapy. In this study, Achberger and colleagues showed efficient transduction of various recombinant adeno-associated viral (AAV) vectors in human ROs and evaluated AAV transduction efficiency using their microfluidic model. It was found that all AAV serotypes demonstrated strong tropism towards Müller glial cells, rod cells, and cone cells. In addition, the microfluidic platform could be used for screening the transduction efficiency of novel AAV variants. To further develop the current model, the addition of a functional choroidal vasculature and the integration of immune cells was discussed. Furthermore, limited by the organizational structure and the nature of the ROs, the authors acknowledged the fact that the current retinal organoid-on-chip lacks the vitreous fluid in which such AAV vectors are usually injected (Achberger et al., 2021).

Another disease model was reported by Su and colleagues, who created ROs and RPE cells from patient iPSCs with a mutation in the USH2A gene associated with retinitis pigmentosa (Su et al., 2022). The USH2A gene encodes for the usherin protein which can bind to proteins of the extracellular matrix and the basal lamina playing a role in development and homeostasis of the retina. The ROs and RPE cells carrying the mutation showed a higher number of apoptotic cells, abnormal protein expression, and abnormal organization of the extracellular matrix. Combining the cellular constructs on a microfluidic chip demonstrated that perfusion itself decreased the number of apoptotic cells and increased the expression of certain extracellular matrix proteins in mutated ROs (Su et al., 2022).

### 2.3 Spinal cord organoids

The spinal cord is an extended and thin nervous tissue that runs through the centre of the spine and is responsible for relaying signals from all over the body to the brain related to body movement, senses, and reflexes. Sensing pain or noxious stimuli is part of the so-called nociceptive circuit. Spinal cord organoids (SCOs) are a recent addition to the field of stem cell-derived organoids able to recapitulate part of the nociceptive circuit, as reviewed by Buchner et al. (Buchner et al., 2023). Although SCOs have great potential for screening new pain therapeutics, standardization of culture procedures and measurement of electrophysiological parameters of these organoids are major challenges.

#### 2.3.1 Spinal cord organoids-on-chip

To measure electrophysiological parameters, Ao et al. created a hPSC-derived spinal organoid-on-a-chip system using a 3D-printed organoid holder with a porous membrane that enabled culturing in an air–liquid interface which was required for maturation. The organoids could then easily be transferred to a 24-wellplate consisting of multiple-electrode array (MEA) sensors (Ao et al., 2022). Gene expression analysis showed that the SCOs contained peripheral sensory neurons and spinal cord interneurons. The system could be used to model nociception as mean firing rate (MFR) increased in response to certain pain-evoking agents. Potential pain treatments were also tested and showed that pro-toxin II (ProTx-II) and tetrahydrocannabinol (THC) effectively reduced MFR when combined with pain-evoking agents (Ao et al., 2022).

#### 2.3.2 Translational potential of spinal cord organoids-on-chip

The translational potential of the same system was further demonstrated by Cai et al. (2023). In their research, the platform was used to model pain relief by stimulating the organoids with capsaicin or by adding an acute opioid to reduce the MFR. Observations in modeling opioid-tolerance and hyperalgesia were found to closely resemble those found in spinal cord slices from animals. Yet, the authors acknowledge that the model has certain limitations, such as the fact that the developed neurons could present a lower maturity level of those found *in vivo* (Cai et al., 2023). Apart from the presented studies focusing on pain therapeutics, the potential of SCOs can be expanded towards studying mechanisms and treatment for spinal cord injuries and developmental spinal cord issues, as reviewed recently by Zhou et al. (2024).

### 2.4 Kidney organoids

Kidney organoids can resemble the *in vivo* tissue in terms of structure and functionality with the formation of both tubular and glomerular compartments (Morizane and Bonventre, 2017; Takasato et al., 2015). Despite their ability to recapitulate some of the key kidney features, their development is limited by the absence of sufficient vascularization (Takasato et al., 2015; Wu et al., 2018; van den Berg et al., 2018). Analyses of the gene expression profiles of tubular epithelial cells and podocytes reveal immaturity of the kidney organoids (Takasato et al., 2015; Wu et al., 2018). The production of sufficiently vascularized and matured organoids has been based on animal transplantation, imposing a burden on the *in vitro* application of kidney organoids (van den Berg et al., 2018; Bantounas et al., 2018).

Kidney organoid-on-chip culture reports mainly addressed the promotion of *in vitro* maturation of kidney organoids, specifically through the enhancement of their vascularization. For example, Homan et al. used millifluidic chips to study the impact of exposure of hPSC-derived kidney organoids to dynamic conditions on their levels of maturation and vascularization (Homan et al., 2019).

#### 2.4.1 Fluidic control enhances kidney organoid development and functionality

Organoids cultured in millifluidic chips under dynamic flow presented increased maturation of tubular compartments, as supported by gene expression analysis, and a higher level of vascularization (Figure 3C2). Conversely, organoids cultured in static conditions presented gene expression profiles similar to those observed in early stages of kidney development during the first trimester. These results were supported by significantly enhanced tubular polarity (Figure 3C1), and an enrichment in primary cilia observed in fluidic conditions. Additionally, the higher degree of tubular maturation was highlighted by the upregulation of drug and epithelial transporters suggesting an increased functional potential of kidney organoids cultured under flow. In addition to this, increased vascular potential under flow conditions was observed via the increased population of endothelial progenitor cells. Notably, the formed networks appeared perfusable with and at a multi-scale level. The heterogeneous expression of melanoma cell adhesion molecule (MCAM), which is a protein that is highly expressed by cellular components of the blood vessel wall, and platelet endothelial cell adhesion molecule (PECAM1), a cell-cell adhesion protein, indicated both immature and mature vascular areas. Consistent with the observed overall enhanced vascularization under fluidic culture conditions, the vascular invasion of podocytes clusters was markedly higher in dynamically cultured organoids compared to those in static conditions. Podocyte foot process maturation was indicated as well, by the formation of more prominent foot process-like structures and the upregulation of foot process related genes and several podocyte adult markers (Homan et al., 2019).

#### 2.4.2 Vascularization of kidney organoids promotes enhanced structural maturation

The effects of fluidic culture on the vascularization of kidney organoids and podocyte maturation were recently confirmed by a similar study where a syringe pump based microfluidic system was used to incorporate kidney organoids (Figure 4C). Nephrin (NHPS1) is a kidney-specific transmembrane protein that plays a critical role in the development of functional mammalian glomerular filtration barriers. Lee et al. reported the increased expression of NHPS1 and PECAM1 regions in kidney organoids cultured on a fluidic chip compared to organoids cultured under static conditions. This observation was consistent with the observation of more prominent foot process-like structures of PECAM1-positive endothelial cell populations under flow conditions (Lee et al., 2021). Another study reported a 2- and 4- fold increase of areas positive for MCAM and PECAM respectively upon on-chip culture (Figure 3C2). In addition, the percentage of MCAM/PECAM co-localization was increased during on-chip conditions, suggesting intermediate maturation levels representative of the *in vivo* endothelial maturation pattern processes. The researchers achieved more extensive kidney organoid vascularization by on-chip seeding human umbilical vein endothelial cells (HUVECs) which subsequently migrated into the organoid structure, forming lumen-like structures interconnected with the endogenous endothelial cell populations (Figure 3C1) (Bas-Cristóbal Menéndez et al., 2022).

#### 2.4.3 Applications of kidney organoids-on-chip in modelling development and drug response

The potential of kidney organoid-on-chip technology to model kidney development was explored by Homan et al. The fluidic culture conditions of kidney organoids cultured in this study led to the formation of glomerular structures similar to those in E14.5 mouse kidneys and raised insights into the mechanisms underlying glomerular vascularization (Homan et al., 2019). Also, drug response prediction studies have been performed using kidney organoid-on chip approaches. In this light, the assessment of nephrotoxicity of a commonly administered immunosuppressive drug (tacrolimus) revealed a significantly higher viability of kidney organoids in static culture conditions compared to organoids cultured under flow. These observations suggest that on-chip organoids may be more sensitive to drugs (Lee et al., 2021).

### 2.5 Liver organoids

Simple organoids that display either hepatocyte or cholangiocyte specific structures and functions have been developed from various sources, including hPSC differentiation (Sampaziotis et al., 2015; Szkolnicka et al., 2014), mature cell transdifferentiation (Huang et al., 2014; Du et al., 2014), and human adult or fetal liver primary cells (Zhang et al., 2018b; Fu et al., 2019; Sampaziotis et al., 2021). Efforts to generate complex liver organoids with a vascular or biliary network consisting of various cell types, have also been reported (Takebe et al., 2017; Stevens et al., 2017). However, zonal structural organization or exocrine functionality have not been reported without *in vivo* engraftment.

Several studies explored the incorporation of liver organoids into microfluidic chips to demonstrate the effects of on-chip culture on organoid maturation and possible applications. A proof-of-concept study identified both hepatic and cholangiocyte cell populations in hPSC-derived organoid structures cultured in a microfluidic chip (Wang et al., 2020). Gene expression profiles revealed the expression of markers related to hepatocyte maturity and drug metabolism. The formation of luminal structures resembling the *in vivo* bile ducts and a pro-longed albumin secretion also indicated structural and functional organoid maturity, respectively (Wang et al., 2020).

#### 2.5.1 Fluidic culture conditions enhance maturation of liver organoids

Both structural and functional maturation have been shown to be induced by fluidic on-chip culture of liver organoids. For example, a study by Teng et al. demonstrated an increased maturation and structural complexity of liver organoids cultured under flow conditions compared to organoids cultured in a static environment (Teng et al., 2021). Fluidic culture conditions led to the formation of organoids with rounder and smoother boundaries. Cortical actin organization appeared more prominent in organoids cultured under flow with the observation of dense actin bundles located close to the organoid surface. In contrast, in static conditions, the actin network showed irregular distribution throughout the organoid. Moreover, organoids formed under flow developed ultrastructural features, such as apical pores and cell-cell junctions, which were absent in static culture. Bile canaliculi and rough endoplasmic reticulum (ER) formation were exhibited in fluidic conditions, while only small vacuo-like structures were observed in static culture conditions. Further, liver-characteristic functional aspects appeared to be more pronounced in dynamic organoid culture. The observed elevated expression of genes encoding for albumin and enzymes involved in drug metabolism suggests an enrichment in mature hepatocytes and thus, higher hepatocyte activity in the liver organoids (Teng et al., 2021). A follow-up study demonstrated that these increased gene expression levels under fluidic conditions translated into secretion of albumin as well as enhanced cytochrome P450 activity in liver organoids cultured in a microfluidic platform compared to the ones cultured statically (Au et al., 2014). In addition, fluidic culture conditions resulted in enhanced cell polarization as indicated by the more localized expression of the apical transporter multidrug resistance-associated protein 2 (MRP2), while in static conditions MRP2 was found to be dispersed throughout the cell (Au et al., 2014). The superior maturity of liver organoids cultured under flow was recently demonstrated by Byeon et al. as well. Their results confirmed the more prominent structure of liver organoids-on-chip as indicated by the expression of MRP2 and zona occludens protein 1 (ZO-1) (Figure 3D1). Increased albumin and CYP genes expression highlighted the increased maturation of liver organoids cultured under flow (Figure 3D2). Interestingly, fluidic culture conditions also enabled the faster formation of liver organoids by reducing the standard static-based differentiation protocol by 12 days (Byeon et al., 2024).

#### 2.5.2 Liver organoids-on-chip for disease modeling and drug testing applications

Extending on above-mentioned physiological observations, Wang et al. exploited liver organoid-on-chip technology to model nonalcoholic fatty liver disease (NAFLD) which is a pathology characterized by excessive fat build-up in the liver. By exposing the liver organoids to free fatty acids (FFA), they were able to recapitulate the major key characteristics of the disease *in vitro*. Specifically, these liver organoids exhibited accumulation of triglycerides and formation of lipid droplets upon treatment. The increased expression of lipid-metabolism associated genes indicated the non-physiological metabolic activity of treated organoids. Additionally, NAFLD-like phenotypical production of reactive oxygen species (ROS) and upregulation of genes involved in inflammatory processes were observed in FFA-treated organoids (Wang et al., 2020). Teng and co-workers demonstrated that a similar FFA-exposed liver organoid-on-chip technology approach closely mimics the temporal reversal of NAFLD observed *in vivo* in patients with lifestyle alterations. They showed that liver organoids exposed to FFA, followed by a FFA treatment removal, presented a 38% reduction of lipid accumulation compared to untreated organoids. The liver organoids-on-chip platform was additionally used to test three common drugs that are clinically administered to NAFLD patients to reduce retention of lipids in the liver, also termed anti-steatosis drugs. Upon exposure to all three drugs, organoids showed reduced lipid accumulation compared to untreated ones (Teng et al., 2021). Another drug testing application of liver organoid-on-chip technology was demonstrated by exposure to acetaminophen (APAP), a drug which is clinically shown to be associated with acetaminophen-induced hepatotoxicity and which may lead to acute liver failure. A dose-dependent APAP-induced hepatoxic effect was observed in organoids cultured in microfluidic chips reflecting the *in vivo* responses of APAP-treated hepatoma cells. The study was facilitated by a platform that was pioneering in its design to be fully automated, allowing for the generation, culture and analysis of 3D organoids within one system (Au et al., 2014). Overall, the examples discussed above underline the potential of the described liver organoids-on-chip systems for modeling hepatic diseases and developing potentially predictive drug screening platforms.

### 2.6 Gastrointestinal organoids

The gastrointestinal (GI) tract comprises all organs stretching from the mouth to the anus that are collectively responsible for the digestion of food, absorption of nutrients, excretion, and protection against, e.g., bacterial invasion, housing an extensive immune system and highly diverse microbiota population (Cheng et al., 2010; Brittan and Wright, 2002). hPSC-derived gastric and intestinal organoids self-organize to recapitulate some of the key features of the GI system such as the distinct cell types present, epithelial structure, and transport and absorptive functions. The organoids’ residing stem cells furthermore have the capacity to self-renew, and to differentiate towards major intestinal lineages (Taelman et al., 2022). One key biophysical feature of both stomach and intestine is the presence of the luminal flow. However, as GI organoids self-organize as closed spherical structures encircling a lumen this posed a challenge. By utilizing the adaptability of microfluidic systems, a microenvironment to facilitate the application of luminal fluid flow to the organoid can be engineered. In that way, processes like metabolic waste and cell debris removal can be enabled, while transport and drug application studies can also be facilitated.

#### 2.6.1 Establishing intraluminal flow by puncturing a three-dimensional organoid

In a pioneering study, Lee et al. combined luminal flow with peristaltic motion in an iPSC-derived human stomach-on-chip (Lee et al., 2018). Their microfluidic device featured a central chamber for culturing the organoid and two in-line chambers for culture media. Gastric organoids were created by following the McCracken protocol established for human intestinal organoid (HIO) differentiation and adapting it to yield a gastric lineage (McCracken et al., 2011). Lee et al. embedded matured single gastric organoids in Matrigel in the central culture chamber. Subsequently, the organoid was pierced on opposite sides by two micropipettes connected to tubing. Luminal flow and peristaltic motion were generated by employing a peristaltic pump. By incorporating a FITC dextran solution the authors demonstrated luminal flow and rhythmic stretch and contraction of the gastric organoid. The platform created in the proof-of-concept study could be used to model gastric diseases and drug delivery. In a follow-up study involving the platform it would be of interest to see the authors address the long-term effects of luminal flow and peristaltic motions on organoid long-term viability.

In a different study, Sidar et al. tackled the issue of fluid flow in intestinal organoids in a similar fashion as Lee and colleagues. Here, they developed a millifluidic device connected to a syringe pump for fluid-flow control (Sidar et al., 2019). The chip was made from acrylic sheets and was composed of three layers with a central organoid well in the center and a top channel to provide extraluminal flow. After differentiation and maturation, the HIO was transferred to the culture chamber and embedded in Matrigel^®^. Intraluminal flow was established by puncturing the organoid with two glass capillaries connected to the pump (Figure 3E1). The authors showed that puncturing did not negatively affect the viability of the organoid and that they could achieve efficient waste clearing from the organoid’s lumen. The intra- and extraluminal fluid flow achieved in the millifluidic chip opens the possibilities to introduce various compounds such as molecules, cellular or bacterial metabolites, drugs or even microbes into the luminal space and follow the interaction with the epithelium. Notwithstanding the advantages offered by the platform, the culture period of 68 h was relatively short and leaves long-term studies suitability to be explored. Moreover, the delicate procedure of puncturing a single organoid does not lend itself easily for high-throughput approaches.

#### 2.6.2 Creation of a physiologically representative crypt-villus intestinal architecture

In an alternative to obtain a perfusable lumen in a HIO, Nikolaev et al., exploited the self-organizing properties of developing organoids and engineered a microenvironment on chip resembling the crypt-villus architecture in the intestine (Nikolaev et al., 2020). For this, their chip consisted of a central hydrogel chamber connected to tubing via a designated in- and outlet. By ablating the hydrogel with a laser, microcavities resembling crypts in the intestinal epithelium were formed. Two external medium reservoirs flanking the organoid chamber provided extraluminal medium. Seeding iPSCs into the scaffold and subsequent differentiation revealed a spatial arrangement of stem cells and epithelial subtypes resembling cell patterning along the crypt-villus axis *in vivo*. Proliferative areas were confined to artificial crypts with offspring cell migration towards the lumen. This was also marked by the classic characteristic of the intestine of the self-renewing epithelial lining, which they further demonstrated in the system’s regeneration capabilities after injury (Figure 3E2a). The authors also addressed the long-term culture potential of their device by demonstrating culture periods of more than 30 days while preserving the tissue’s spatial organization. In an approach to model host-microorganism interactions, the authors infected the gut-tubes with *Cryptosporidium parvus* (Figure 3E2b), demonstrating the parasite’s full life cycle and sustained growth over 4 weeks. During this infection, the tissue retained its integrity and allowed for detailed stage identification and host immune responses.

#### 2.6.3 3D organoid integration vs. organoid-derived 2D intestinal epithelia on-chip

While this review focusses on the advancements in integrating whole organoids in microfluidic systems, an alternative approach in using organoid-derived 2D epithelia to replicate the gastrointestinal system on chip is becoming more and more prevalent and merits mentioning. 2D epithelia derived from dissociated mature organoids are often grown on porous membranes under flow conditions which presents advantages regarding transport studies, high-throughput applications, and imaging (Moerkens et al., 2024; Workman et al., 2018). This also opens the possibility of host-microbe interaction studies as epithelia are more readily accessible (Jalili-Firoozinezhad et al., 2019). However, the loss of the organoids’ intrinsic spatial organization and cell patterning, as well as complex 3D multi-tissue interactions in these 2D cultures remains a significant drawback.

### 2.7 Multi-organoid-on-chip approaches

The culture of individual organoids and tissues on micro- and millifluidic chips paved the way to gaining insights into drug responses, embryonic development and molecular and signaling pathways. Recent research activities exploited the potential of simultaneous incorporation of different types of organoids into a single chip device designed to establish physiologically and anatomically representative connections between these organoids facilitating studies into inter-organ interactions. Apart from the potential to investigate inter-organ communication beyond what is currently known, various studies have focused on developing platforms aimed at recapitulating drug interactions at a systemic level.

#### 2.7.1 Multi-organoid-on-chip platforms for drug metabolism and toxicity

Yin et al. used a chip platform to co-culture liver organoids and heart spheroids (Yin et al., 2021). After generation of hPSC-derived cardiac spheroids in the bottom chamber of the chip with added micropillars to avoid organoid fusion, generated liver organoids were transferred to the top microwell chamber. Communication between the two tissue types was facilitated by placement of a porous membrane positioned between the two chambers. Organoids exhibited functional features as indicated through the synthesis of urea and albumin by liver organoids and the contraction/beating of the cardiac spheroids. This liver-cardiac co-culture system was used to assess the cardiotoxic effect of the antidepressant drug clomipramine after its metabolism through the liver. The liver organoids could metabolize clomipramine, as indicated by the expression of enzymes involved in drug metabolism, and by mass spectrometry-based detection of the respective metabolites. In the presence of clomipramine, cardiac spheroids co-cultured with liver organoids showed reduced viability and decreased cardiac beating and beating velocity compared to cardiac spheroids cultured in absence of liver organoids. These observations suggested that the observed cardiotoxic effects of clomipramine were liver metabolism dependent (Yin et al., 2021). Skardal et al. demonstrated the ability of a heart-liver-platform to predict *in vivo* drug responses of both toxic and non-toxic compounds (Skardal et al., 2020). The system correctly distinguished between liver and cardiac toxicity effects induced by drugs recalled from market by the Food and Drug Administration (FDA) from non-toxic pharmacological reagents (Skardal et al., 2020).

To enable prediction of a rare cardiovascular complication upon administration of the cytostatic compound bleomycin to treat an ovarian germ cell tumor (Didagelos et al., 2013), Skardal et al. extended on the liver-heart platform by adding a lung module (Figures 3F1, 4D) (Skardal et al., 2017). Exposure to bleomycin resulted in a decreased contraction rate of cardiac spheroids co-cultured with liver and lung modules compared to cardiac monocultures (Figure 3F2). Also, exposure of lung epithelium to bleomycin led to the production of inflammatory factor interleukin-1β in mono-as well as co-cultures, which subsequently acted as a secondary cardiotoxic factor (Skardal et al., 2017).

Skardal et al. further evolved their previously reported multi-organ-on-chip approach by working towards a body-on-a-chip platform. The chip was composed of several microfluidic chips with organoid-chambers in one single device. The connection was made using tubing and culture medium was pumped by a micro-peristaltic pump which permitted the simultaneous culture of heart, liver, testis, colon/brain organoids and lung and vascular elements. The system exhibited retained cellular viability upon culture for 28 days. The body-on-chip platform was used to assess the toxic effects of two cancer prodrugs on healthy tissues. It was revealed that the liver critically drives toxicity of these compounds as a toxic response was selectively observed in multi-organ chips containing liver organoids. These liver organoids metabolized these prodrugs into their toxic active forms (Skardal et al., 2020). A similar approach was used by Rajan et al. to study organ exposure to the chemotherapeutic prodrug ifosfamide. Upon combining liver, heart, brain and testes organoids with lung and blood vessel constructs, ifosfamide exposure was shown to reduce the viability of brain organoids but only in the presence of liver organoids on chip (Rajan et al., 2020).

These studies highlight that modular multi-organ on chip models provide a potent approach to facilitate inter-organ communication and discern the distinct contributions of individual organs to drug activity and toxicity. This enables researchers to methodically dissect and analyze the complex interplay among various organs, shedding light on the impact of drugs on diverse physiological systems and facilitating the development of better targeted and safer pharmaceuticals.

#### 2.7.2 Multi-organoid-on-chip platforms for modeling health and disease

The application of multi-organoid-on-chip systems for disease modeling is increasingly explored. Tao et al. used microwells combined with microfluidic channels connected to a peristaltic pump to enable co-culture liver and islet organoids with the aim to mimic liver-islet interaction under both healthy and type 2 diabetes mellitus (T2DM) conditions (Tao et al., 2022). On-chip co-culture resulted in enhanced viability, maturation and functionality of both organoid types. Hepatic and pancreatic maturation markers were upregulated and there was a notable increase in the secretion of albumin and insulin under co-culture conditions while recapitulating islet-liver functional coupling. Gene enrichment analysis revealed differential regulation of metabolic pathways upon co-culture. In addition, the system replicated the insulin-stimulated glucose uptake process. Islet organoids exhibited glucose-stimulated insulin secretion, while liver organoids showed significant increase in glucose consumption in the co-culture setting. To mimic T2DM, hyperglycemic conditions were induced via high glucose concentrations (25 mM) in the medium. These led to decreased spare respiratory capacity and adenosine triphosphate (ATP) production in both organoid types, reminiscent of the mitochondrial dysfunction clinically observed in T2DM patients (Schmid et al., 2011). During hyperglycemia, protein levels of glucose transporter 1 were reduced in liver organoids and the expression of their respective encoding genes was downregulated as well matching clinical reports (Chadt and Al-Hasani, 2020). These observations demonstrated the system’s ability to recapitulate early stages of T2DM in an islet-liver axis. Treatment with metformin, which targets glucose transporter proteins to regulate cellular glucose metabolism, rescued the hyperglycemia-induced phenotype for both islet and liver organoids, indicating the platform’s potential for T2DM drug development in a multi-organ scale (Tao et al., 2022).

Another recent application of multi-organoid-on-chip development was reported by Nguyen et al. by combining kidney tubuloids and liver organoids in the commercially available TissUse HUMIMIC Chip2 (TissUse GmbH, Germany) microfluidic platform (Nguyen et al., 2022). This model was used to mimic the therapeutic effect and systemic biodistribution of extracellular vesicles (EVs). EVs, small lipid-bilayer particles (<200 nm), carry various cell-originating biomacromolecules like nucleic acids and proteins. Target cells can uptake EVs, influencing their behavior, sparking therapeutic interest (Wiklander et al., 2019). Nguyen and colleagues induced acute kidney injury through hydrogen peroxide exposure which led to compromised epithelial barrier function of kidney tubuloids and decreased multidrug resistance-associated protein 2 function. Administration of mesenchymal stem cell-derived EVs rescued the injured tubuloids. Accumulation of EVs was 2.6-fold higher in the injured tubuloids compared to the uninjured ones. Notably, EVs were also detected in the liver compartment in both cases of injured and in non-injured kidney tubuloids, with a 2.1-fold increase in accumulation in the former one (Nguyen et al., 2022). These reports collectively demonstrate the value of incorporating organoids representing a diversity of tissue types, enabling the study of organ-organ interactions, drug- or gene-mediated multi organ pathologies, and potential therapeutic investigations.

## 3 Challenges for organoids-on-chip technology

### 3.1 Biological challenges of organoid-on-chip culture

While the unique potential and application potential of organoids has been solidly demonstrated, a major biological challenge lies within their heterogeneity. Organoids self-organize from stem cells to recapitulate essential structural features of organs and feature various cell types (Takebe and Wells, 2019). Due to the innate stochastic nature that characterizes the self-organization process of organoids, cell type content, their proportion and spatial distribution are subject to variability. This is further amplified by the use of different protocols across different laboratories impacting on the reproducibility of the obtained results (Hofer and Lutolf, 2021; Hernández et al., 2022). Another challenge of conventional organoid culture is the long-term viability and stability of organoids. During the growth process, organoids naturally undergo structural changes which can lead to changes in stability and long-term viability. However, extended culture may be required to achieve the necessary degree of maturation to facilitate the study into mechanisms and underlying causes in age-related disorders. For example, in cerebral organoids, some gene signatures related to age-associated neurodegenerative diseases are only detected past 250 days (about 8 months) in culture (Gordon et al., 2021).

Recent scientific efforts to combine organoids with microfluidic technology to generate organoids-on-chips led to multiple advancements to overcome some of the limitations of conventional organoid culture. For example, as described in this review, on-chip organoid culture generally promotes extended organoid growth (Berger et al., 2018; Homan et al., 2019), increases structural and functional organoid maturation (Homan et al., 2019; Berger et al., 2018; Lee et al., 2021), and allows for better representative *in vitro* modeling of organ development and disease (Berger et al., 2018; Homan et al., 2019; Lee et al., 2021; Cho et al., 2021; Schuster et al., 2020) (Table 1).

**TABLE 1:** Overview of the main conventional organoid culturing challenges and key organoid-on-chip findings and applications.

Organoid type	Challenges in conventional culture	On-chip findings	Applications	Selected references
Brain	• Insufficient nutrient and oxygen supply limits the extended organoid culture and growth	• Higher and more homogeneous intra-organoid oxygen levels • Larger volume size, prolonged culture life, reduced apoptosis, and higher proliferation rate	• Insights in underlying mechanisms of gyrification during brain development • Modelling of prenatal nicotine exposure • Parkinsons’s disease modelling • Brain organoid vascularization	Berger et al. (2018), Cho et al. (2021), Karzbrun et al. (2018), Wang et al. (2018b), Salmon et al. (2022)
	• Lack of biomechanical stimulation, matrix-cell and cell-cell interactions	• Culture under fluidic conditions in combination with brain extracellular matrix (BEM)		
	• Limited maturation	• Higher gene expression levels of neuron markers • More defined structural organization • Larger brain ventricle-like fluid filled cavities • Higher electrophysiological functionality		
Retina	• Limited maturation	• Enhanced retinal ganglion cell (RGC) development (RGC subtypes enrichment, increase axon outgrowth)	• Insights in RGC development • Drug toxicity screening of anti-malaria drug and antibiotics • Gene therapy model to evaluate AAV transduction efficiency • Disease modelling of retinitis pigmentosa	Gong et al. (2023), Achberger et al. (2021), Su et al. (2022)
	• Absence of a monolayer of retinal pigment epithelium	• Co-culture with RPE cells enhanced the maturation of organoids (increased formation of inner and outer photoreceptor segments and rhodopsin invasion)		
Spinal cord	• Need for electrophysiological activity monitoring	• Integration of chip system with multiple-electrode array (MEA) enabling electrophysiological measurements	• Nociception modelling • Drug testing of pain treatments • Pain relief modelling	Ao et al. (2022), Cai et al. (2023)
Liver	• Limited structural organization	• Rounder and smoother organoid boundaries • More prominent cortical actin organization • Ultrastructural features (apical pores, cell-cell junctions), bile canaliculi and rough endoplasmic reticulum (ER) development	• Modelling of non-alcoholic fatty liver disease (NAFLD) • Anti-steatosis drug screening for NAFLD • Drug hepatoxicity screening	Wang et al. (2020), Teng et al. (2021), Au et al. (2014), Byeon et al. (2024)
	• Limited exocrine functionality	• Increased gene expression of hepatocyte markers • Increased drug metabolism marker activity and hepatocyte marker secretion		
Gastrointestinal tract	• Absence of laminar flow	• Introduction of intraluminal flow through organoid puncturing • Facilitation of intraluminal metabolic waste and cell debris removal	• Modelling of host-microorganism interaction • Modelling of regeneration processes	Sidar et al. (2019), Lee et al. (2018), Nikolaev et al. (2020)
	• Limited structural architecture	• Peristaltic motion mimicking • Engineered microenvironment resembling native organ architecture		
Kidney	• Lack of vascularization	• Enhanced vascularization (increased maturation of endogenous endothelial populations, perfusable network formation, increased podocyte vascular invasion)	• Insights in mechanisms underlying glomerular vascularization • Drug nephrotoxicity screening	Bas-Cristóbal Menéndez et al. (2022), Homan et al. (2019), Lee et al. (2021)
	• Limited maturation	• Increased expression of proximal tubule markers and enhanced tubular polarity • Enrichment in primary cilia • Podocyte foot process maturation		
Multi-organ	• Lack of systemic organoid connection	• Microfluidic connection between different organoid types recapitulating *in vivo* systemic organ-organ interaction • Functional coupling	• Insights in organ-organ communication • Systemic disease (e.g., type 2 diabetes mellitus (T2DM)) • Drug screening at multi-organ level • Studying biodistribution of therapeutic agents	Yin et al. (2021), Rajan et al. (2020), Skardal et al. (2017), Skardal et al. (2020), Tao et al. (2022)

Microfluidic flow facilitates continuous supply of growth factors compared to cyclic spikes with manual organoid medium refreshment. Apart from supply of growth factors at a homogeneous concentration over time, employing gradients of growth factors can steer organoid spatial organization more effectively and in a better controlled manner (Park et al., 2019). Adjusting the perfusion to fit the organoids’ needs ensures optimal nutrient and oxygen supply, and continuous removal of waste products, influencing the long-term viability, stability and functional maturation of the organoids as showcased (Cho et al., 2021; Su et al., 2022; Sidar et al., 2019). However, while microfluidic chips can mimic blood flow, a key challenge is the lack of physiologically relevant vascularization. In the absence of a functional vascular network, organoids face limitations in terms of their size, longevity, and ability to mimic the *in vivo* intricate metabolic and transport processes. Moreover, functional vasculature is relevant for pharmacological applications, for example, when investigating blood-brain barrier permeation of therapeutics and enables the study of diseases that involve vascular components, such as cancer. Recent studies involving brain and kidney organoids showed that the integration of a vascular network in organoid-on-chip cultures significantly improved organoid viability and functional maturation (Salmon et al., 2022; Homan et al., 2019; Lee et al., 2021; Bas-Cristóbal Menéndez et al., 2022; Zhao et al., 2021).

Achieving *in vitro* vascularization of organoids has been a field of strong research interest throughout the last years. Various approaches have been developed, including co-culture of organ-specific organoids with several endothelial cell types or vascular organoids, co-differentiation of organ-specific and vascular organoids, and implementation of 3D/4D bioprinting techniques. Although progress in the field has permitted the generation of organoids with integrated vascular networks, the functionality of the vasculature generated is still debatable (Nwokoye and Abilez, 2024; Werschler et al., 2024). Static-based organoid vascularization models permit cell-cell interactions between the organoid and vascular components; however, they lack vascular network perfusion. Functional vascularization highly depends on the fluidic delivery of nutrients and oxygen to tissues. At the same time, mechanical stimulation holds a key role in vascular network’s physiological development, maturation and function (Rademakers et al., 2019). Organ-on-chip technology offers the possibility to further advance vascularization of organoids by introducing the key element of flow. The chip microchannels are usually lined with endothelial cells creating vessel-like structures subsequently perfused to introduce luminal flow in the system. Recent research examples have demonstrated the formation of interconnected perfusable vascular networks integrated into the organoid tissue supporting its growth and maturation (Nwokoye and Abilez, 2024; Boylin et al., 2024). A major advantage of addressing organoid vascularization through organ-on-chip systems is based on the ability to precisely control the vascular flow parameters applied at a microscale level. This enables the *in vitro* simulation of vascular flow patterns characteristic of specific organs or disease conditions. Besides promoting vascular network functionality, organoid-on-chip vascularization holds great promise for drug testing studies. Drug administration and distribution can be performed through the generated vasculature network rather than direct exposure of the entire organoid tissue, resulting in a process more closely reflecting the *in vivo* conditions. Worth mentioning, latest research paradigms have led to a series of advancements, including recapitulation of complex vascular architectures reaching single capillary resolution (Nguyen et al., 2025), blood and plasma perfusion possibilities (Vatine et al., 2019) and formation of vascular networks interconnecting multiple organoids (Quintard et al., 2024). During the next years, further advancements in the field are anticipated, by combining artificial intelligence (AI) and machine learning (ML) methods to predict optimal vessel formation patterns that better resemble native organotypic vasculature architectures.

Another challenge in conventional organoid culture is the lack of a representative biophysical microenvironment, impacting on their development and maturation. Organoid-on-chip models can recreate a relevant microenvironment, either by incorporating extracellular matrices (Cho et al., 2021; Wang et al., 2018a; Nikolaev et al., 2020), or by designing the chip such that it can provide mechanical cues, such as actuation or shear stress (Homan et al., 2019; Lee et al., 2018; Sidar et al., 2019), achieving a higher degree of physiological relevance and functional maturation in the organoids. Engineering the microenvironment can also accommodate cell-cell and organ-organ interactions, by allowing for the integration of multiple cell or organoid types to achieve cultures that can reflect more complex responses as showcased by several studies discussed in this perspective (Homan et al., 2019; Lee et al., 2021; Salmon et al., 2022; Su et al., 2022; Achberger et al., 2019; Gong et al., 2023; Bas-Cristóbal Menéndez et al., 2022; Yin et al., 2021; Skardal et al., 2020).

### 3.2 Technical challenges in organoid-on-chip culture

One of the major technical challenges in organoid culture is reproducibility. Given that organoids-on-chip is a recent technology that has emerged mainly over the last 5 years, most of the studies focusing on incorporating organoids into chip platforms have been conducted as proof-of-concept studies. As a result, most research work is either limited to demonstrating the feasibility of the culture of an individual or a combination of organoids in microfluidic devices, while largely neglecting a thorough demonstration of their reproducible generation. As mentioned above, organoid differentiation naturally results in degrees of heterogeneity (Hofer and Lutolf, 2021; Hernández et al., 2022). These variations are further amplified by differences in the origin of the cells, such as primary tissue, commercially available embryonic or induced hPSCs, or patient-derived hPSCs (Mohammadi et al., 2021; Hernández et al., 2022). Among the few studies addressing this issue, Cho and co-workers showed improved reproducibility of organoid maturation upon microfluidic culture in two different induced hPSC lines and passages (Cho et al., 2021). In contrast, Berger et al. reported a cell line-dependent increase in midbrain dopaminergic neuron differentiation observed under the fluidic culture conditions of hMO (Berger et al., 2018). The number of passages, culture conditions, and media composition and storage can all contribute to variability in the culture of organoids. Hence, to facilitate reproducibility of data it is important to generate and follow guidelines and standards in (stem) cell culture practice (Pamies et al., 2022).

#### 3.2.1 Reducing variability with defined and animal-free compounds

Another factor adding to variability in experimental outcomes is the tendency to rely on animal-derived compounds, such as fetal bovine serum or basement membrane mixes such as Matrigel^®^ and Geltrex™ for the culture of organoids. These poorly defined compounds contribute to low data reproducibility through their inherent batch-to-batch variability. Culturing organoids using synthetic or better-defined compounds increases their suitability for clinical translation. Nevertheless, currently available animal-free extracellular matrix alternatives require fine-tuning of the material’s mechanical and biological properties as they are known to affect organoid differentiation and growth (Cassel de Camps et al., 2022). Nonetheless, in recent years substantial advancements have been made in utilizing animal product alternatives to deliver technically and ethically improved *in vitro* models (Duarte et al., 2023).

#### 3.2.2 Consensus and standardization of methods needed for future advancements

Greater data reproducibility may be further facilitated by reducing the variability caused by the large diversity of experimental procedures used to generate organoid-on-a-chip systems. The use of different protocols for generating, growing, and maturing various organoid types, along with the utilization of chips with varying features and fabrication designs, imposes a burden on achieving reproducibility. Striving towards a standardized consensus on the specific experimental parameters and functional assays will facilitate the consistent generation of results of higher fidelity. To achieve standardization in the domain of organ-on-chip, multiple consortia have been established. The consortium for safety assessment using human IPS cells (CSAHI) works towards standardizing drug safety evaluations using human PSC-derived cardiomyocytes with advanced platforms to better predict human-relevant cardiotoxicities. To achieve this, CSAHI establishes and distributes standardized protocols for the generation, differentiation and analysis of iPSC-derived cardiomyocytes, conducts strict quality control at every stage and provides labs with well-defined, standardized materials such as cell-lines and assays. Moreover, inter-laboratory studies are conducted regularly to assess the reproducibility obtained with their protocols and materials, as well as offering training and resources to researchers and technicians (Takasuna et al., 2020). The IQ MPS consortium works in a similar fashion, addressing standardization and implementation of gut, liver and kidney organs-on-chips in drug development (Tomlinson et al., 2024). Recently, a consortium has emerged to promote standardization in the construction, evaluation and application of bone organoids (Wang et al., 2025). A major step towards technical reproducibility and standardization of chip fabrication among various labs has also been made recently with the establishment of an ISO standard for microfluidic chips (ISO 22916:2022). In the future, such technical standards could also be applied to include the wide variety of bio- or electrical sensors that are integrated in microfluidic systems for continuous culture monitoring and the detection of target analytes, such as biomarkers, metabolites or pathogens, as reviewed by Buttkewitz et al. (Buttkewitz et al., 2023).

#### 3.2.3 Automated and high-throughput platforms

While this review highlights the value and pioneering innovation of early organoid-on-chip systems, these devices often accommodated only one or a few organoids per chip and relied on manual handling for EB or organoid transfer and media changes (Cho et al., 2021; Wang et al., 2018a; Salmon et al., 2022; Achberger et al., 2019; Homan et al., 2019; Sidar et al., 2019), which introduces variability and compromises the reproducibility of results. An alternative route to achieve reduced variability includes the development of chip platforms designed to facilitate automated and high-throughput (HT) organoid culture through precise control of culture conditions and enabling systematic parallelized testing of various conditions.

Many articles emphasize the high-throughput capabilities of organoid culture devices, however, there is currently no consensus on what constitutes “high-throughput” in the context of organoid creation. In the studies reviewed in this article, the number of organoids generated per HT platform ranges from approximately 20 (Seiler et al., 2022; Zhu et al., 2023) to around 160 (Zhu et al., 2017; Ao et al., 2020; Teng et al., 2021) and even up to 240 at a time. Several platforms also highlight their scalability, including compatibility with multi-well plates, including 384-well formats (Shrestha et al., 2024; Ao et al., 2021). With an outlook on future advancements and model scalability, we would therefore classify these platforms as high*er*-throughput systems.

Next to readout and screening experiments, some of these HT platforms also enable complete organoid differentiation workflows from initial EB aggregation to maturation, thereby eliminating the necessity for manual EB transfer. The efficiency and consistency of EB aggregation were shown to be influenced by the architecture of the organoid compartment, which can be optimized utilizing micropillars in microarray platforms (Zhu et al., 2017; Wang et al., 2020) or microwells (Drabbe et al., 2025; Teng et al., 2021).

An emerging automated method for reproducible, efficient and high-throughput tissue and organoid creation is bioprinting. In this technique, cells, biomaterials and growth factors are printed and arranged in three-dimensional structures to create complex, functional and biomimetic tissues (as reviewed by Chand et al. (Chand et al., 2025). For example, acoustic forces have been used to create up to 6,000 tumor organoid aggregates (Chen et al., 2019). Another bioprinting approach employs droplet-based microfluidics to encapsulate stem cells in biocompatible hydrogel microcapsules that provide a controlled size and environment for organoid growth and function (Liu et al., 2020; van Loo et al., 2023). Zhu et al. applied this method generate homogenous EBs, and to create organoid assembloids to produce region-specific human brain organoids in a highly controlled and higher-throughput manner (Zhu et al., 2023). Acoustic aggregation and microencapsulation were also shown to facilitate efficient and homogeneous cell aggregation when used in concert (Wang et al., 2024).

Bioprinting was also recently used by Shin et al. to create functional kidney organoids where controlled seeding density significantly influenced physiologically relevant organoid patterning and maturation (Shin et al., 2024). Shrestha et al. combined automated bioprinting with a microarray platform fitted to a 384-well plate to create and culture human liver organoids. Analysis after 25 days showed comparable morphology and superior functionality of bioprinted organoids compared to those conventionally cultured, and demonstrated their suitability for *in situ* toxicity assessments (Shrestha et al., 2024).

Automated HT platforms can not only improve the throughput of organoid creation but also reduce variability introduced by manual handling through precise control of the cell aggregation and culture conditions, such as fluid flow, temperature, pressure and shear forces. Compared to conventional methods, organoids grown and produced by HT platforms often showed improved morphology and function that can be attributed to the controlled initial formation, enhanced perfusion and minimized shear forces. However, many high-throughput platforms feature only short to mid-length experimental time frames allowing only preliminary conclusions about long-term culturing (Seiler et al., 2022; Zhu et al., 2017; Drabbe et al., 2025; Zhu et al., 2023; Shrestha et al., 2024). Downstream analysis of long-term cultures is needed to address concerns such as hypoxic core formation and other culture challenges.

Another limitation of HT platforms is the absence of compartmentalization between organoids, which hinders individual assessment of organoid responses, such as metabolite or neurotransmitter screenings. While Seiler et al. (Seiler et al., 2022) developed an automated platform with individually addressable culture chambers, it still required manual transfer of pre-formed EBs to the culture device. Microfluidic large-scale integration (mLSI) chips offer a potential solution by combining high-throughput, automation and individually addressable chambers. MLSI chips emerged as a clear paradigm of automated, high-throughput platforms. These systems contain hundreds to thousands of individually addressable culture chambers (Gómez-Sjöberg et al., 2007; Zhang et al., 2019). Vollertsen et al. demonstrated the simultaneous operation of three different mLSI systems at the same time through a fluidic circuit board, enabling a highly parallelized culture in microfluidic devices (Figure 4E) (Vollertsen et al., 2020). Advances in 2D mLSI systems have utilized liquid handling robotics with custom multiplexed microfluidic devices designed in a well-plate footprint (Novak et al., 2020; Azizgolshani et al., 2021; Winkler et al., 2022). Recently, also 3D mLSI platforms have been developed to culture samples with increased diameter sizes, thus facilitating automated and high-throughput culture of 3D tissue constructs, including spheroids and organoids (Ko et al., 2024; Ardila Riveros et al., 2021) and showcased by Au et al., to improve reproducibility (Au et al., 2014).

Despite their relevance and future perspective, high-throughput systems face their own set of significant challenges and limitations (Probst et al., 2018). Overall, addressing the challenge of reproducibility demands the combination of different disciplines to enable the development of standardized, advanced, engineered organoid-on-chip platforms and the unified effort of scientific community to create a consensus on their use and methodologies.

## 4 Outlook for organoids-on-chip technology

### 4.1 Drug discovery

The field of drug discovery can be facilitated in terms of both speed and cost by using organoids-on-chip platforms. Drug discovery generally constitutes a complex, long process where human organoids are particularly valuable during the early pre-clinical phases of target identification and validation, while more complex organs-on-chips could be valuable in the final pre-clinical stages of drug efficacy and safety evaluation (Rossi et al., 2018; Esch et al., 2015). The integration of organoids into chip platforms can result in a single well-defined system suitable for application along the drug discovery pipeline combining the advantages of both biotechnological and pharmaceutical research. Particularly, multi-organoids-on-chip systems are relevant tools for investigating drug metabolism, pharmacodynamics, pharmacokinetics and targeting, as the effects of drugs are not solely determined by the action of a single organ but are the result from complex interactions within multiple organs (Skardal et al., 2020; Skardal et al., 2017; Rajan et al., 2020). These processes can be comprehensively explored and visualized in anatomically and physiologically connected multi-organoid microfluidic platforms. The relevance of organoids-on-chips in drug discovery has recently been validated by the FDA modernization act in 2011 now allowing alternative methods for drug and biologics testing, such as organoids and organoids-on-chips, to be used for regulatory approval processes in an aim to reduce animal testing.

### 4.2 Personalized medicine

Another area significantly benefiting from organoids-on-chip technology is personalized medicine. The application of patient-derived organoids in personalized medicine is often limited by the lack of environmental factors necessary for reflecting the complete *in vivo* disease-specific phenotype (Drost and Clevers, 2017), which could be facilitated by on chip culture (Bhatia and Ingber, 2014; Zhang et al., 2018a). Patient-derived organoids-on-chip systems combine the genetic and phenotypic background of the patient enabling their use as personalized drug response models.

### 4.3 Regenerative medicine

Another application for organoids-on-a-chip lies in regenerative medicine. Tissue regeneration upon organoid transplantation has been demonstrated in animal models (Schutgens and Clevers, 2020). The clinical translation of the approach requires the further functional maturation of organoids to increase chances of proper engraftment and sufficient function in the human organism, which is facilitated by on-chip culture. Although many challenges still must be addressed, organoids-on-chip technology can take regenerative medicine one step closer to the ambitious goal of tissue and organ regeneration.

## 5 Conclusion

The synergistic engineering of organoids and microfluidic chip technology promises a new scientific era combining the advantages of two powerful bioengineering tools for *in vitro* modeling of human biology. Future interdisciplinary research work will establish the innovative technology of organoids-on-chip and will open new avenues in the fields of drug discovery, developmental biology, personalized and regenerative medicine.
